# From Cells to Plaques: The Molecular Pathways of Coronary Artery Calcification and Disease

**DOI:** 10.3390/jcm13216352

**Published:** 2024-10-23

**Authors:** Andreas Mitsis, Elina Khattab, Evi Christodoulou, Kimon Myrianthopoulos, Michael Myrianthefs, Stergios Tzikas, Antonios Ziakas, Nikolaos Fragakis, George Kassimis

**Affiliations:** 1 Cardiology Department, Nicosia General Hospital, State Health Services Organization, Nicosia 2029, Cyprus; andymits7@gmail.com (A.M.); khattab_elina@outlook.com (E.K.); kimonmyr@yahoo.com (K.M.); myr.michael@shso.org.cy (M.M.); 2 Cardiology Department, Limassol General Hospital, State Health Services Organization, Limassol 3304, Cyprus; evi.christodoulou@yahoo.com; 3 Third Department of Cardiology, Aristotle University of Thessaloniki, 54636 Thessaloniki, Greece; tzikas@gmail.com; 4 First Department of Cardiology, AHEPA University Hospital, Aristotle University of Thessaloniki, 54636 Thessaloniki, Greece; aziakas@auth.gr; 5 Second Department of Cardiology, Aristotle University of Thessaloniki, 54642 Thessaloniki, Greece; fragakis.nikos@gmail.com

**Keywords:** coronary artery calcification (CAC), coronary artery disease (CAD), atherosclerosis, vascular calcification, inflammation, vascular smooth muscle cells (VSMCs), plaque stability, osteogenic differentiation, molecular pathways

## Abstract

Coronary artery calcification (CAC) is a hallmark of atherosclerosis and a critical factor in the development and progression of coronary artery disease (CAD). This review aims to address the complex pathophysiological mechanisms underlying CAC and its relationship with CAD. We examine the cellular and molecular processes that drive the formation of calcified plaques, highlighting the roles of inflammation, lipid accumulation, and smooth muscle cell proliferation. Additionally, we explore the genetic and environmental factors that contribute to the heterogeneity in CAC and CAD presentation among individuals. Understanding these intricate mechanisms is essential for developing targeted therapeutic strategies and improving diagnostic accuracy. By integrating current research findings, this review provides a comprehensive overview of the pathways linking CAC to CAD, offering insights into potential interventions to mitigate the burden of these interrelated conditions.

## 1. Introduction

Coronary artery calcification (CAC) is increasingly recognized as a critical component in the development and progression of coronary artery disease (CAD), the leading cause of morbidity and mortality worldwide [1]. CAC is a complex, multifaceted process characterized by the deposition of calcium in the coronary arteries, leading to obstructive CAD [2]. This calcification not only serves as a hallmark of advanced atherosclerosis but also plays a direct role in the pathophysiology of CAD, contributing to plaque stability and the risk of acute coronary events [3].

Understanding the pathophysiological mechanisms linking CAC and CAD is essential for several reasons. First, CAC is a strong and independent predictor of cardiovascular events, making it a valuable marker for assessing CAD risk. Second, the molecular and cellular processes driving CAC are closely linked with those that promote atherosclerosis, including inflammation, lipid accumulation, and smooth muscle cell proliferation. These processes contribute to the formation and progression of calcified plaques, which can significantly impact the clinical course of CAD [4]. Despite advancements in imaging modalities and treatments, the cellular and molecular pathways contributing to CAC remain incompletely understood. This review aims to fill this gap by exploring the complex interplay of molecular signals that drive CAC, thereby providing a foundation for novel therapeutic interventions.

This review aims to provide a comprehensive overview of the current understanding of the molecular pathways involved in CAC and its relationship with CAD. By exploring the roles of vascular smooth muscle cells (VSMCs), inflammatory cytokines, lipid metabolism, and genetic factors, we seek to elucidate the complex interactions that underlie plaque calcification. Additionally, we will discuss the clinical implications of CAC, including its role in risk stratification, diagnostic imaging, and potential therapeutic interventions.

## 2. Methods

This narrative review is based on a comprehensive literature search conducted in databases such as PubMed, Google Scholar, and Scopus. Keywords such as ‘coronary artery calcification’, ‘atherosclerosis’, ‘molecular pathways’, and ‘vascular smooth muscle cells’ were used to identify relevant peer-reviewed articles. Studies were selected based on relevance to the topic, publication date, and citation count. The search strategy aimed to include the most recent and impactful studies to ensure the review’s thoroughness. Cross-referencing of key articles was conducted to minimize the possibility of missing critical data.

## 3. Cellular and Molecular Mechanisms of Coronary Artery Calcification

CAC is characterized by the calcium phosphate deposition process in various conditions, such as atherosclerosis, hypertension [5], aortic valve stenosis, CAD, diabetes mellitus (DM) [6], chronic kidney disease (CKD) [7], hyperlipidemia [8], and chronic inflammatory disorders. CAC, intimal or medial, stiffens arteries and weakens vulnerable atherosclerotic plaques, increasing the risk of rupture [9]. Intimal calcification is frequently associated with damage and dysfunction of endothelial cells (EC), as well as atherosclerosis [10]. It is caused by the imbalance of the vascular microenvironment, where the pro-calcific factors (inflammation, endoplasmic reticulum (ER) stress, mitochondrial dysfunction, iron homeostasis, programmed cell death (PCD), and other cellular metabolic dynamics) are enhanced [11,12,13].

VSMCs play a key role in the development of CAC, and the loss of VSMCs through various forms of PCD, such as apoptosis, necrosis, necroptosis, pyroptosis, autophagy, and ferroptosis, contributes to the thinning of fibrous caps and the formation of necrotic cores, leading to calcification [14,15]. Recent studies have highlighted nucleotide-binding and oligomerization domain-like receptor family pyrin domain-containing 3 (NLRP3)-mediated pyroptosis as a protective mechanism, while autophagy has been identified as a key process in diabetes-related calcification [16]. Autophagy can inhibit CAC by suppressing the osteogenic differentiation of VSMCs through mechanisms involving pathways such as anti-differentiation non-coding RNA, b-catenin, and AMP-activated protein kinase (AMPK), while it may also promote calcification via cAMP response element-binding protein signaling, elastin degradation, and long non-coding RNA H19-mediated extracellular signal-regulated kinase (ERK) signaling [17,18,19,20].

Phosphoglycerate dehydrogenase (PHGDH) inhibits CAC in VSMCs by preventing ferroptosis via the P53/solute carrier family 7a member 11 (SLC7A11) pathways, making it a therapeutic target [21]. Indoleamine 2,3-dioxygenase 1 (IDO1) deficiency promotes calcification through enhanced Runt-related transcription factor 2 (RUNX2) activity, while kynurenine, an IDO1 catabolite, counteracts this by promoting RUNX2 degradation via aryl hydrocarbon receptor (AhR) [22]. Low C1 tumor necrosis factor-related protein 3 (CTRP3) levels are linked to CAC in diabetic patients, as CTRP3 inhibits osteogenic differentiation in VSMCs by blocking β-catenin nuclear translocation [23]. Nϵ-carboxymethyl-lysine (CML), an advanced glycation product, promotes calcification in diabetic atherosclerosis by enhancing foam cell formation [24]. Inhibitors like fetuin-A, matrix Gla protein (MGP), and osteoprotegerin (OPG) prevent calcium deposition [25,26,27]. Epidermal growth factor receptor (EGFR) inhibition reduces calcification by blocking calcifying vesicle release from VSMCs, while sortilin regulates calcification via Rab11 trafficking [28]. Osteopontin (OPN) and matrix metalloproteinase-9 (MMP-9) contribute to CAC and atherosclerosis progression, with MMP-9 enhancing macrophage infiltration and matrix degradation [29]. CML promotes calcification in diabetic VSMCs through the CML/RAGE-ROS-p38MAPK-cbfα1-ALP pathway [30].

The Wingless-type family member (Wnt)/β-catenin signaling pathway promotes CAC, particularly in response to high phosphorus levels [31]. Wnt3a, Wnt5a, and Wnt5b are upregulated in human coronary plaques, influencing VSMC differentiation, calcification, and cholesterol handling [32]. This pathway stimulates RUNX2, a key transcription factor in the osteogenic transformation of VSMCs [33,34]. In high-phosphate environments, β-catenin translocases to the nucleus, inducing RUNX2 expression and driving calcification, particularly in end-stage renal disease (ESRD) patients [34]. High phosphate also regulates Pit-1 transcription via this pathway, accelerating CAC [35]. Deleting peroxisome proliferator-activated receptor gamma (PPARγ) promotes vascular calcification through a Wnt5a-driven chondrogenic pathway, while PPARγ protects against calcification by inducing the Wnt antagonist secreted frizzled-related protein 2 (sFRP2) [36]. Osteocalcin (OCN) and low-density lipoprotein receptor-related protein 8 (LRP8) further enhance this process, while Moscatilin inhibits vascular calcification by suppressing the Wnt/β-catenin pathway [37].

Lipoprotein a (Lp(a)) promotes CAC by increasing calcific deposits, alkaline phosphatase (ALP) activity, and pro-calcific proteins like bone morphogenetic protein 2 (BMP2) and OPN [38]. Lp(a) also stimulates VSMC mineralization and endothelial-to-mesenchymal transition (EndMT), which contributes to calcification via the Notch1-BMP2-Smad1/5/9 signaling pathway [39]. Inhibition of Notch1 reduces BMP2 expression, ALP activity, and calcification [40]. Lp(a) also activates the nuclear factor-κB (NF-κB) pathway, enhancing inflammation by upregulating OPN and cytokines like interleukin (IL) IL-1β and IL-6 [38]. Clinical studies link higher Notch1 and OPN levels to CAC and show Lp(a) associated with increased CAC volume in individuals with inflammation [41,42]. Finally, adiponectin inhibits osteoblastic differentiation in VSMCs via the AMPK pathway, while indoxyl sulfate promotes CAC by suppressing Notch signaling [43], and notch1-Msx2 interactions play a key role in early-stage vascular calcification, independent of BMP-2 and RUNX2 pathways [44,45].

## 4. Inflammation and Coronary Artery Calcification

Chronic inflammation, driven by factors like oxidative stress, cytokines (e.g., C-reactive protein [CRP], Tumor necrosis factor-α (TNF-α), and interleukins (IL-1β, IL-6, IL-18), plays a key role in CAC and age-related diseases [46]. Pro-inflammatory cytokines (e.g., IL-6, IL-8, TNF-α, monocyte chemoattractant protein-1 [MCP-1]) activate pathways that promote VSMC osteogenic transformation [47,48,49]. IL-18 enhances CAC via the ERK1/2 pathway, while TNF-α and IL-6 upregulate VSMC calcification through the AP-1/c-FOS pathway [50]. IL-29 also promotes CAC via JAK2/STAT3 signaling, increasing BMP2 expression [51]. Although some studies link inflammatory markers to CAC, their association is weak after adjusting for traditional risk factors [52]. Additionally, ER stress contributes to CAC by promoting extracellular vesicle release and interacting with iron homeostasis and mitochondrial dysfunction [53]. Lower serum levels of netrin-1 and gremlin-1, alongside higher TNF-α, may serve as early diagnostic markers for subclinical CAC [54].

**Figure 1:**
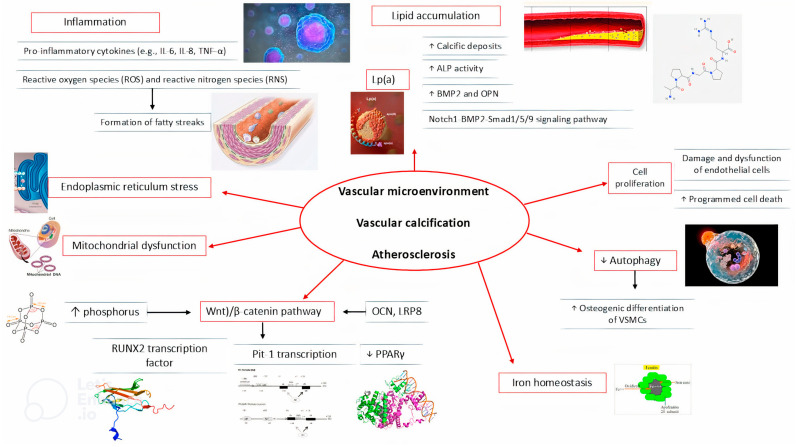
The mechanistic pathways leading to coronary artery calcification.

Atherosclerotic plaque calcification is regulated by the receptor activator of the nuclear factor-kappa B ligand (RANKL)/OPG system, affecting both calcium deposition and plaque stability [55]. Key pathways like Notch, Wnt, and TGF-β/BMP control osteogenic differentiation and inflammation, involving macrophage activation and VSMC proliferation [56]. Ferroptosis and calcification are interconnected via Toll-like receptor 4 (TLR4) and the NF-κB axis [57]. Tetramethylpyrazine (TMP) reduces CAC by inhibiting caspase-3/GSDME-mediated pyroptosis, lowering inflammation, calcification, and oxidative stress in VSMCs [58]. Clinical studies reveal that macrophage-rich plaques have a higher calcification burden, making them more vulnerable, highlighting that inflammation in conditions like rheumatoid arthritis and non-alcoholic fatty liver disease increases CAC risk [59,60]. Of note, elevated interleukin-2 receptor (IL-2R) levels in CAD patients and IL-6 in CKD are linked to CAC severity, underscoring chronic inflammation’s role in CAC development and the need for early diagnosis [61,62] (Figure 1).

This figure illustrates the complex interplay between cellular mechanisms, inflammatory pathways, and molecular signaling contributing to coronary artery calcification (CAC). Key processes include lipid accumulation, endothelial cell dysfunction, and vascular smooth muscle cell (VSMC) osteogenic differentiation. These pathways are driven by the upregulation of pro-inflammatory cytokines, oxidative stress, and the activation of transcription factors such as RUNX2 and Wnt/β-catenin. Collectively, these processes result in the deposition of calcific material within the vascular wall, contributing to the development and progression of atherosclerotic plaques.

ALP: Alkaline Phosphatase; BMP2: Bone Morphogenetic Protein 2; CAC: Coronary Artery Calcification; IL: Interleukin; Lp(a): Lipoprotein(a); LRP8: LDL Receptor-Related Protein 8; OCN: Osteocalcin; OPN: Osteopontin; PPARγ: Peroxisome Proliferator-Activated Receptor Gamma; ROS: Reactive Oxygen Species; RNS: Reactive Nitrogen Species; RUNX2: Runt-Related Transcription Factor 2; TNF-α: Tumor Necrosis Factor Alpha; VSMC: Vascular Smooth Muscle Cells; Wnt: Wingless/Integrated signaling pathway.

## 5. Lipid Accumulation and Smooth Muscle Cell Proliferation

Atherogenesis is a chronic inflammatory process triggered by the interaction between circulating lipoproteins and subendothelial extracellular matrix molecules, specifically proteoglycans, leading to the retention of lipoproteins. Low-density lipoprotein (LDL) plays a central role, along with other apoB-containing lipoproteins smaller than 70 nm in diameter, in contributing to the formation of atherosclerotic plaques [63]. These plaques contain cholesterol, fatty substances, cellular waste products, calcium, and fibrin [64].

LDL particle concentration (LDL-P) subtypes, along with large high-density lipoprotein (HDL)-P subtypes, have been associated with CAC progression. However, after adjusting for standard risk factors and traditional lipid measurements—such as LDL cholesterol (LDL-C), HDL cholesterol (HDL-C), and triglycerides—only the medium and very small LDL-P subtypes remained significantly related to CAC progression. CAC was more prevalent in individuals with either low HDL-C or the highest HDL-P levels [65]. Additionally, elevated lipoprotein(a) [Lp(a)] levels are widely known to increase cardiovascular risk and contribute to CAC development [66,67].

According to the response-to-retention atherosclerosis model, atheroma development in the vessel wall is driven by a complex inflammatory process [68]. Reactive oxygen species (ROS) and reactive nitrogen species (RNS) oxidize lipids and LDL [69]. Oxidized LDL, concentrated in the intimal layer of the vessel, plays a crucial role in initiating and advancing atherosclerosis by damaging endothelial cells, enhancing leukocyte adhesion, and influencing the production of leukocyte and monocyte adhesion molecules (e.g., VCAM, ICAM, E-selectin, and P-selectin) in the endothelium [70,71]. This damage promotes the infiltration of the intimal vessel layer by monocytes, T cells, and mast cells [72].

Through the action of MCP-1, M-CSF, and IL-8, monocytes differentiate into macrophages, which phagocytose oxidized LDL using scavenger receptors (SR-A family) and transform into lipid-rich foam cells [73]. While foam cells were traditionally thought to originate from bone marrow-derived macrophages, recent studies suggest that vascular smooth muscle cells (VSMCs) can also convert into foam cells when exposed to aggregated or oxidized LDL. In fact, the majority of foam cells in atherosclerotic plaques may derive from VSMCs [74,75].

The accumulation of foam cells in the arterial wall leads to the formation of fatty streaks [76]. VSMCs migrate from the media to the intima, proliferating to form fibrous atherosclerotic plaques from these fatty streaks [77]. As atherosclerosis progresses, proteolytic enzymes such as metalloproteinases—produced by macrophages and T-lymphocytes—destabilize the fibrous plaque. Damage to the fibrous cap and collagen results in coagulation, thrombus formation, and ultimately, occlusion of the arteries [78]. Table 1 provides a summary of all the molecular pathways involved in CAC (Table 1).

## 6. Genetic and Environmental Factors in CAC and CAD

As in many complex diseases, the risk of developing CAC and CAD is modulated by a set of genetic and environmental factors. Accessibility to human genome sequencing enables the performance of genome-wide association studies and meta-analyses studies supporting the strong genetic component that drives CAC and CAD events [82]. More than 160 loci have been associated with the risk of developing CAD. Interestingly, a great percentage of these variants are not associated with the traditional pathogenesis pathways of CAD such as lipid metabolism, blood pressure, inflammation, extracellular matrix function and structure, and vascular remodeling [83,84,85], supporting the existence of undiscovered pathological mechanisms. From these studies, only four loci (9p21, PHACTR1/EDN1, APOE, and APOB) have been associated with CAC [86]. Recent studies confirmed the association of these four loci with CAC and identified new loci that are implicated in VSMC calcification, bone mineralization, phosphate, and vitamin metabolism [87,88].

Additionally, rare variants association studies enable the identification of rare mutations in different genes which influence the risk of CAD. Inactivating mutations in LDLR, LPL, and APOA5 are associated with increased risk while mutations in PCSK9, NPC1L1, ASGR1, APOC3, ANGPTL4, and LPA are associated with decreased risk of CAD [89,90,91]. These genes are implicated in pathways related to LDL, triglyceride-rich lipoproteins, cholesterol, or lipoprotein metabolisms [92]. Arterial calcification is also connected with rare mutations in different genes. The related genes can be divided into three groups: (1) genes associated with extracellular purine/phosphate/phosphate metabolism such as ENPP1, ABCC6, PDGFRB, SLC20A2, XPR1, MYORG, and LMNA; (2) genes associated with interferonopathies such as IFIH1 and DDX58; and (3) the GBA gene which is associated with Gaucher disease, a known lysosomal storage disorder characterized by increased levels of glucosylsphingosine and glucosylceramide in various organs [93,94,95,96,97]. Further clinical studies deciphering the molecular mechanisms of these mutations would lead to the development of novel therapeutic options for CAC and CAD.

Not only genetic predisposition but also various environmental factors play a role in the development of CAC and CAD. The European Society of Cardiology and the American College of Cardiology guidelines state cholesterol, blood pressure, cigarette smoking, diabetes, and adiposity are the major risk factors for coronary disease [98]. Age, gender, and ethnicity have been shown to play a role in the onset of CAC [99,100]. The Multi-Ethnic Study of Atherosclerosis (MESA) study has shown, by measuring coronary calcification, that whites have the greatest CAC, followed by Chinese, Hispanics, and African Americans [101]. Other studies, such as the Heinz Nixdorf Recall [102] and Coronary Artery Risk Development in Young Adults (CARDIA) studies [103] have shown a positive correlation between smoking and CAC onset. Cigarette smoking through the production of toxic chemicals causes vascular calcification [104]. Additionally, the relationship between physical activity and CAC remains controversial. While the CARDIA study had shown that physical activity reduces the risk of having CAC [105], the MESA study failed to show this correlation [106]. Studies in athletes have shown that participants with exercise volume > 2000 MET-min/wk have a significantly higher CAC score [107]. Recently, the MARC-2 study suggests that exercise intensity but not volume is associated with coronary artery calcification progression [108]. Studies on favorable cardiovascular health highlight the importance of a healthy lifestyle and early prevention to preserve a healthy cardiovascular state and reduce the risk of developing CAD later in life [109].

## 7. Clinical Implications and Diagnostic Approaches

The management of CAC and its implications for CAD requires a comprehensive approach that includes early detection, advanced diagnostic methods, and the identification of potential biomarkers [110,111]. Early detection of CAC is crucial for effective risk stratification and patient management [112]. By identifying CAC at an early stage, clinicians can better assess the risk of CAD-related events and implement preventive measures [113]. Monitoring the progression of CAC over time allows for timely interventions that could slow the advancement of atherosclerosis, potentially reducing the incidence of major cardiovascular events [114]. Furthermore, regular monitoring aids in adjusting the intensity of preventive strategies, such as lifestyle modifications or pharmacological treatments, to better manage the patient’s condition [115,116].

### 7.1. Imaging Modalities for the Detection of CAC

A variety of imaging modalities are available for detecting and quantifying CAC, each with its own advantages and limitations. Cardiac multi-slice computed tomography (MSCT) imaging remains the most commonly used imaging modality [117]. Cardiac CT is promoted by both the European Society of Cardiology [118] and the American Heart Association [119] for the non-invasive evaluation of patients with low-to-intermediate risk for CAD. Cardiac CT can be used either for the detection of CAC or for the precise visualization of coronary arteries. Coronary computed tomography angiography (CTA) is often favored over plain coronary artery calcium assessment because it not only quantifies the degree of stenosis in the coronary arteries but also provides a qualitative assessment of plaque morphology by visualizing both calcified and non-calcified plaques, assessing their characteristics, and directly measuring the severity of arterial narrowing [120].

CAC with MSCT can be assessed by various scoring systems. The three primary methods for assessing coronary artery calcification by CT are the CT Calcium Scoring (CACS), Calcium Volume Score, and Calcium Mass Score. The CACS, or Agatston score, is widely used and provides a simple, standardized measure of calcified plaque burden but focuses only on calcified plaques and can be influenced by image noise [121]. The CACS offers a valuable measure of calcified plaque burden and helps predict cardiovascular risk [122]. This method is highly predictive of future cardiovascular events, making it a valuable tool in clinical practice [123]. The Calcium Volume Score assesses the total volume of calcified plaques, offering a more comprehensive evaluation and greater stability over time, though it is less commonly used and requires more detailed processing [124,125]. Finally, the determination of the calcium mass score is another option for the quantification of CAC [126]. The Calcium Mass Score combines plaque volume and density, providing a physiologically relevant measure of plaque burden that may offer better risk stratification; however, it is more complex and less widely adopted in clinical practice. Each method has unique advantages and disadvantages, making them suitable for different clinical scenarios depending on the goals of the assessment [127].

The Coronary Artery Calcium Data and Reporting System (CAC-DRS) provides a standardized framework for reporting CAC scores derived from CT scans, which are crucial in assessing the risk of cardiovascular events, particularly CAD. CAC-DRS categorizes CAC scores into four distinct groups: 0 (indicating no detectable coronary calcium), 1–99 (reflecting mild plaque buildup), 100–299 (indicating moderate calcium levels and more significant plaque), and ≥300 (suggesting extensive calcium deposits and a high risk of cardiovascular complications) [128,129]. This stratification allows healthcare providers to accurately gauge a patient’s risk and tailor prevention and treatment strategies accordingly. A higher CAC-DRS category is associated with a greater likelihood of cardiovascular events, prompting more intensive management [130]. By ensuring consistent and clear reporting of CAC scores, CAC-DRS enhances clinical decision-making and supports more effective risk assessment and intervention for patients at varying levels of cardiovascular risk [131].

CAC detection using positron emission tomography (PET)/CT scans offers a powerful combination of anatomical and functional imaging, enhancing the assessment of CAD [132]. The CT component provides high-resolution images for detecting and quantifying calcified plaques, while the PET component adds functional insight by identifying metabolically active or inflamed plaques that may not yet be calcified but pose significant risk [133]. This hybrid approach improves diagnostic accuracy and offers enhanced risk assessment by capturing both calcified and non-calcified plaques, making it particularly valuable in high-risk patients or those with complex coronary anatomy [134]. However, PET/CT is more costly, less widely available, and involves higher radiation exposure compared to standard CT, which may limit its use to specialized centers or select patient populations [135]. Despite these limitations, PET/CT’s ability to integrate anatomical and metabolic information provides a comprehensive tool for guiding treatment decisions and monitoring disease progression in CAD.

Cardiac magnetic resonance imaging (CMR) is not typically utilized for the detection of CAC due to its limited sensitivity to calcium deposits [136]. CMR excels in providing detailed assessments of cardiac structure and function, offering valuable information on myocardial tissue characterization, including the detection of fibrosis, edema, and infarction. Additionally, CMR is highly effective in evaluating ventricular function, wall motion abnormalities, and myocardial perfusion, particularly under stress conditions, making it an important tool for diagnosing and managing various aspects of CAD [137]. CMR offers the advantage of avoiding ionizing radiation, making it a safer option for some patients. However, CMR does not play a direct role in detecting calcified plaques within the coronary arteries, it serves as a complementary modality alongside CT imaging by offering comprehensive insights into the functional and structural implications of CAD on the heart [138].

Intravascular imaging modalities, such as intravascular ultrasound (IVUS) and optical coherence tomography (OCT), can provide additional insights into plaque activity and coronary artery wall structure [139], though they are less frequently used for routine CAC assessment due to their invasiveness and cost. IVUS offers deeper penetration, making it effective for visualizing the entire vessel wall and assessing the overall plaque burden, including the quantification of calcification [140]. However, it has a lower spatial resolution, which may miss finer details like micro-calcifications. On the other hand, OCT provides much higher resolution, allowing for detailed visualization of plaque morphology and precise delineation of calcified plaques, though it has limited penetration depth and requires blood clearance during imaging [141]. The choice between IVUS and OCT depends on the specific clinical scenario, with IVUS being better for assessing deeper or more extensive calcifications, while OCT is superior for high-resolution imaging of plaque details. Often, these modalities are used complementarily to provide a comprehensive assessment of coronary artery disease.

In developing a comprehensive diagnostic approach for CAC, it is essential to integrate the various imaging modalities by assessing their strengths according to the specific clinical scenario (Table 2). For instance, CTA and CACS are often first-line choices for non-invasive evaluation due to their accessibility and ability to provide both anatomical and, in the case of CTA, functional insights. PET/CT can be reserved for high-risk patients or those with complex coronary anatomy, where the combined anatomical and metabolic information can guide more nuanced therapeutic decisions. Intravascular modalities like IVUS and OCT, while more invasive, are particularly valuable during interventional procedures where detailed plaque characterization is critical for optimal stent placement. Patient characteristics play a crucial role in modality selection; for example, CMR might be preferred in patients requiring frequent imaging due to its lack of ionizing radiation, while renal function considerations may influence the use of contrast-enhanced studies like CTA. Ultimately, the choice of imaging modality should be tailored to the patient’s risk profile, clinical needs, and specific diagnostic or therapeutic goals, ensuring a personalized and effective approach to cardiovascular care.

### 7.2. Biomarkers for the Detection of CAC

The search for reliable biomarkers to enhance risk assessment for CAC and CAD is an ongoing area of research. Inflammatory markers such as CRP, soluble intercellular adhesion molecule- 1 (sICAM-1), and fibrinogen have been associated with increased CAC and may serve as indicators of active disease [142]. CRP is significantly associated with CAC progression among clinical parameters [143] and can be used to predict the risk of an elevated coronary artery calcium score [144]. However, a recent large meta-analysis did not show the significant role of CRP in risk stratification for CAC scores [145]. IL-6 has been found to be associated with the progression of CAC in patients with chronic renal dysfunction on dialysis [146]. IL-6 is positively associated with vascular calcification, promoting inflammation and contributing to the progression of calcification in vascular tissues [147].

Lipid-related biomarkers, including LDL-C and ApoB, are central to the process of atherosclerosis and could help predict the likelihood of calcification [148,149]. The atherogenic index of plasma (AIP), an indicator calculated based on TG levels and HDL-C levels, has been proposed to assess the degree of CAC, showing greater predictive power for atherosclerosis and cardiovascular events [150]. AIP has been found to be an independent predictor of CAD and has been used as a risk factor for CAC and CVD [151,152].

Genetic markers, particularly specific polymorphisms, are also being investigated for their potential to identify individuals at higher risk for CAC [153]. Recently, it has been described that genetic polymorphisms on the NPC1L1 gene were associated with high-degree CAC in male patients with premature CAD [154]. Additionally, emerging biomarkers like MGP, which inhibits vascular calcification, adiponectin which is inversely associated with vascular calcification, suggesting a protective role against the progression of calcification in vascular tissues, and OPG, which is involved in bone metabolism, are being studied for their roles in vascular health and may offer new opportunities for assessing CAC risk [155]. Low plasma adiponectin levels [156] and elevated plasma OPG [157] are associated with the progression of CAC and aortic plaque, while MGP has been proposed as a major factor in the development of vascular calcification [158]. Fetuin-A is a glycoprotein that plays a crucial role in inhibiting calcium deposition in blood vessels [159]. Lower levels of Fetuin-A are associated with increased vascular calcification, making it a potential biomarker for assessing cardiovascular risk [160]. Finally, BMPs, and especially BMP-2, are key regulators of bone formation and are also involved in the pathological process of vascular calcification [79]. BMP-2 promotes the osteogenic differentiation of vascular smooth muscle cells, leading them to adopt bone-like properties and contribute to the deposition of calcium in the arterial walls. Elevated levels of BMP-2 have been linked to increased calcification, making it an important biomarker and potential therapeutic target in cardiovascular disease [80].

The integration of early detection with advanced imaging techniques and biomarker research represents a comprehensive approach to managing CAC and CAD. By combining these strategies, clinicians can better identify high-risk patients, monitor disease progression, and tailor interventions to prevent adverse cardiovascular outcomes. The continued exploration of these diagnostic approaches and biomarkers is essential for enhancing the overall management of CAD associated with CAC, ultimately improving patient care and outcomes.

## 8. Therapeutic Strategies Targeting CAC

Pharmacological strategies for managing CAC primarily focus on reducing the progression of calcification through lipid-lowering agents, anti-inflammatory drugs, and agents that modulate mineral metabolism [161]. These interventions aim to stabilize plaques, reduce cardiovascular risk, and potentially slow the calcification process (Table 3). Calcium channel blockers (CCBs) and renin-angiotensin system (RAS) inhibitors play significant roles in modulating vascular calcification. CCBs, by preventing calcium influx into VSMCs, help reduce the cellular processes that contribute to calcification, potentially slowing the progression of arterial stiffness [162,163]. RAS inhibitors, such as ACE inhibitors and angiotensin II receptor blockers (ARBs), reduce the effects of angiotensin II, a key driver of vascular calcification through its promotion of inflammation, oxidative stress, and osteogenic differentiation of vascular smooth muscle cells [164,165]. By inhibiting these pathways, both CCBs [166,167] and RAS inhibitors [168,169] offer therapeutic potential in reducing vascular calcification and the associated cardiovascular risks, particularly in patients with hypertension or chronic kidney disease.

Statins are the cornerstone of pharmacological therapy for CAC due to their dual action of significantly lowering LDL-C levels and exerting potent anti-inflammatory effects, both of which are crucial in reducing the progression of calcification [170]. By decreasing LDL-C, statins help reduce the lipid core of atherosclerotic plaques, thereby stabilizing them and preventing further calcification. Moreover, their anti-inflammatory properties play a key role in mitigating the inflammatory processes that contribute to vascular calcification resulting in plaque stabilization [171]. Furthermore, statins can modulate the calcification of VSMCs, providing further evidence of their beneficial effects beyond cholesterol reduction [172]. Clinical studies have demonstrated that statin therapy is associated with a slower progression of calcified plaque burden, underscoring their importance in managing CAD [173,174,175]. These findings collectively reinforce the critical role of statins in not only managing lipid levels but also in directly influencing the progression of CAC, making them a fundamental component of CVD management.

Anti-inflammatory drugs, such as colchicine, are gaining attention for their potential role in reducing CAC by targeting the underlying inflammatory processes that contribute to plaque instability and calcification [176]. Colchicine, traditionally used for gout, has been shown in recent studies to reduce cardiovascular events [177,178], potentially by mitigating inflammation within the arterial walls [179]. In addition to anti-inflammatory agents, novel therapies targeting specific pathways involved in calcification, such as PCSK9 inhibitors and RANKL inhibitors, are also being explored. PCSK9 inhibitors, initially developed to lower LDL cholesterol levels, have shown potential in reducing coronary plaque volume and may also play a role in limiting calcification within the arterial walls. Inhibitors like evolocumab and alirocumab, by reducing LDL and lipoprotein(a), can mitigate calcific progression [180,181]. Additionally, calcification inhibitor therapies are promising areas of exploration. These interventions target molecular pathways involved in osteogenic differentiation of VSMCs, such as the Wnt/β-catenin and BMP signaling pathways [182]. RANKL inhibitors, which interfere with the osteogenic pathways that drive vascular calcification, offer a promising approach to directly targeting the calcification process [81]. In addition to traditional therapeutic strategies, natural compounds such as resveratrol have shown promise in preventing atherosclerosis progression. For instance, Sirasanagandla et al. demonstrated that maternal resveratrol supplementation ameliorates bisphenol A-induced atherosclerotic lesion formation in offspring, suggesting that dietary intake of foods rich in resveratrol could reduce future cardiovascular risk [183]. These emerging therapies represent a significant advancement in the potential treatment options for patients at risk of CAC, offering hope for more effective management strategies in the future.

**Table 3:** Therapeutic Approaches Targeting Calcification Pathways.

Therapeutic Approach	Target Pathway	Mechanism of Action	Potential Clinical Impact
Statins [170,171,172,173,174,175]	Lipid metabolism	Reduces cholesterol and inflammation, indirectly inhibiting calcification	Lower cardiovascular event risk
PCSK9 Inhibitors [180,181]	Lipid metabolism	Lowers LDL cholesterol, potentially influencing calcification	Reduces plaque progression and improves outcomes
Vitamin K [184,185]	Calcium metabolism	Activates MGP to inhibit vascular calcification	Prevents progression of calcified plaques
RANKL Inhibitors [81]	Osteogenic pathway	Blocks osteoclast differentiation, limiting calcification	May reduce the progression of coronary artery calcification
Omega-3 Fatty Acids (EPA) [186,187]	Inflammation	Suppresses inflammatory pathways and inhibits calcification	Potential role in reducing coronary artery calcification

EPA: Eicosapentaenoic Acid; LDL: Low-Density Lipoprotein; MGP: Matrix Gla Protein; PCSK9: Proprotein Convertase Subtilisin/Kexin Type 9; RANKL: Receptor Activator of Nuclear Factor Kappa-Β Ligand.

Lifestyle interventions, including regular physical activity, smoking cessation, and a heart-healthy diet rich in fruits, vegetables, and whole grains, are essential components of CAC management [188]. Dietary modifications, particularly those reducing saturated fats and increasing omega-3 fatty acids, can lower inflammation and lipid levels, indirectly influencing calcification progression [186]. Specifically, eicosapentaenoic acid (EPA), an omega-3 polyunsaturated fatty acid found in fatty fish and fish oils, has been reported to directly inhibit arterial calcification [187]. Vitamin K supplementation plays a crucial role in the modulation of vascular calcification as it is essential for the activation of MGP and the overall stimulation of osteoblastogenesis [189]. Supplementing with Vitamin K, particularly in individuals with low dietary intake or those at risk for vascular calcification, may help reduce the progression of calcification and improve cardiovascular outcomes [184]. Finally, magnesium supplements may be associated with less vascular calcification [185]. Of note, increased dietary calcium intake has not been associated with an increased risk of CAC, suggesting that calcium consumed through diet does not contribute to the development of calcified plaques in the coronary arteries [190,191]. All the aforementioned anti-arterial calcification therapies could be valuable not only as preventive measures for the general population but also particularly beneficial for patients with low bone turnover conditions, such as those with osteoporosis or a significant number of individuals with CKD.

Future therapeutic strategies for CAC are likely to focus on more targeted interventions that specifically inhibit the molecular pathways responsible for calcification. One promising area of research involves the development of therapies that target osteogenic differentiation in VSMCs [192,193,194]. This process, where VSMCs undergo a transformation into osteoblast-like cells, is a key driver of vascular calcification [195]. Inhibitors of this differentiation process could potentially prevent or reduce calcification, offering a more direct approach to managing CAC. For instance, research into inhibitors of the BMP pathway, which is heavily involved in osteogenic differentiation, is showing potential in preclinical studies [196].

Additionally, advancements in precision medicine are paving the way for more personalized therapeutic approaches. By leveraging genetic and biochemical profiling, future therapies could be tailored to an individual’s specific risk factors and underlying biological mechanisms driving calcification [197]. For example, individuals with specific genetic polymorphisms that influence calcium metabolism, or inflammatory pathways could benefit from customized treatments that target these specific processes. Precision medicine also holds promise in identifying patients who may respond particularly well to novel therapies, such as PCSK9 inhibitors or RANKL inhibitors, further optimizing the management of CAD and CAC [198,199].

These approaches, supported by ongoing research and clinical trials (NCT05720156, NCT04889053, NCT05482399, and NCT05259046) suggest a future where the treatment of CAC is not only more effective but also highly individualized, addressing the unique needs of each patient based on their genetic and molecular profiles [200]. As our understanding of the molecular mechanisms underlying CAC deepens, these targeted and personalized therapies are expected to play a crucial role in the prevention and treatment of coronary artery disease.

## 9. Conclusions

CAC is a complex process driven by multiple molecular and cellular pathways, including inflammation, oxidative stress, and osteogenic differentiation of vascular smooth muscle cells. As a significant contributor to CAD, CAC not only serves as a marker of advanced atherosclerosis but also plays a critical role in plaque stability and progression. Current research efforts are aimed at understanding the intricate mechanisms that regulate calcification and exploring therapeutic interventions to slow or reverse this process. The integration of advanced imaging techniques and personalized medicine offers new hope for risk stratification and tailored treatment strategies. As the molecular underpinnings of CAC continue to be uncovered, innovative therapeutic approaches targeting specific calcification pathways could lead to better clinical outcomes in CAD patients.

While this review aims to provide a comprehensive overview of the molecular mechanisms underlying CAC, there are inherent limitations. The narrative nature of the review may result in selection bias, as studies with inconclusive or negative results may be underrepresented. Additionally, emerging molecular pathways not yet extensively studied could present new therapeutic targets. Future research should focus on large-scale, multi-center studies that validate the clinical applicability of the pathways discussed here. Investigating the role of novel compounds, such as anti-inflammatory agents and calcification inhibitors, in clinical trials would be a valuable next step in targeting CAC.

## Figures and Tables

**Table 1:** Molecular Pathways Involved in Coronary Artery Calcification.

Pathway	Key Molecular Players	Role in Calcification
Inflammatory Pathways	IL-1β [46], TNF-α, NF-kB [47,48,49]	Promote VSMCs differentiation and osteogenic transformation, contributing to calcified plaques
Oxidative Stress Pathway	ROS, NADPH oxidase [69]	Enhances inflammation and calcification through oxidative damage and VSMCs differentiation
Osteogenic Pathways	BMPs [79,80], RANKL [55,81], RUNX2 [33,34]	Induce VSMCs transformation into osteoblast-like cells, driving calcification
Autophagy	AMPK, mTOR, Beclin-1, b-catenin [17,18,19,20]	Dysregulated autophagy promotes calcification by increasing osteogenic differentiation

AMPK: AMP-activated protein kinase; BMP: Bone Morphogenetic Protein; IL-1β: Interleukin 1 Beta; mTOR: Mechanistic Target of Rapamycin; NF-kB: Nuclear Factor Kappa-light-chain-enhancer of activated B cells; RANKL: Receptor Activator of Nuclear Factor Kappa-Β Ligand; ROS: Reactive Oxygen Species; RUNX2: Runt-related transcription factor 2; TNF-α: Tumor Necrosis Factor Alpha; VSMCs: Vascular Smooth Muscle Cells.

**Table 2:** Comparison of Imaging Modalities for Coronary Artery Calcification (CAC) Detection.

Imaging Modality	Pros	Cons	Key Characteristic
Cardiac Multi-Slice CT (MSCT) [118,119]	Most common modality for CAC detection	Focuses mainly on calcified plaques	Broad availability and relatively low cost of this modality.
	Supported by the European Society of Cardiology and the American Heart Association	Can be influenced by image noise	
	Non-invasive evaluation of coronary arteries	Higher radiation exposure	
	Quantifies degree of stenosis	Requires the use of contrast agents	
CT Calcium Scoring (CACS) [122,123]	Simple, standardized measure of calcified plaque burden	Only assesses calcified plaques	Important role in routine clinical practice for risk stratification.
	Highly predictive of future cardiovascular events	Can be affected by image noise	
Calcium Volume Score [124,125]	Assesses total volume of calcified plaques	Less commonly used	Useful for tracking changes in plaque burden over time.
	Offers greater stability over time	Requires more detailed processing	
Calcium Mass Score [126]	Combines plaque volume and density	More complex to calculate	More valuable in research settings or for detailed patient evaluations.
	Provides a physiologically relevant measure	Less widely adopted in clinical practice	
	Potentially better for risk stratification		
PET/CT scans [132,133,134,135]	Combines anatomical and functional imaging	More costly	Important role in guiding therapeutic decisions, especially in complex cases.
	Enhances diagnostic accuracy by identifying metabolically active plaques	Less widely available	
	Particularly valuable in high-risk patients	Higher radiation exposure	
Cardiac Magnetic Resonance Imaging (CMR) [136]	Provides detailed assessments of cardiac structure and function	Limited sensitivity to calcium	Important role as a complementary modality, especially for patients requiring frequent imaging without radiation exposure.
	No ionizing radiation	Not typically used for detecting calcification	
	Effective for evaluating myocardial tissue		
Intravascular Ultrasound (IVUS) [139,140]	Deeper penetration into vessel wall	Lower spatial resolution	Invasive method typically reserved for specific clinical scenarios or interventional procedures.
	Effective for assessing overall plaque burden	May miss fine details like micro-calcifications	
	Useful for quantifying calcification	Invasive procedure	
Optical Coherence Tomography (OCT) [141]	High spatial resolution	Limited penetration depth	Important role in pre-intervention planning, particularly when fine detail is crucial.
	Detailed visualization of plaque morphology	Requires blood clearance	
	Precise delineation of calcified plaques	Invasive and costly	

## Data Availability

Data are contained within the article.
